# FYPO: the fission yeast phenotype ontology

**DOI:** 10.1093/bioinformatics/btt266

**Published:** 2013-05-08

**Authors:** Midori A. Harris, Antonia Lock, Jürg Bähler, Stephen G. Oliver, Valerie Wood

**Affiliations:** ^1^Cambridge Systems Biology Centre and Department of Biochemistry, University of Cambridge, Sanger Building, 80 Tennis Court Road, Cambridge CB2 1GA, UK and ^2^Department of Genetics, Evolution & Environment and UCL Genetics Institute, University College London, Darwin Building, Gower Street, London WC1E 6BT, UK

## Abstract

**Motivation:** To provide consistent computable descriptions of phenotype data, PomBase is developing a formal ontology of phenotypes observed in fission yeast.

**Results:** The fission yeast phenotype ontology (FYPO) is a modular ontology that uses several existing ontologies from the open biological and biomedical ontologies (OBO) collection as building blocks, including the phenotypic quality ontology PATO, the Gene Ontology and Chemical Entities of Biological Interest. Modular ontology development facilitates partially automated effective organization of detailed phenotype descriptions with complex relationships to each other and to underlying biological phenomena. As a result, FYPO supports sophisticated querying, computational analysis and comparison between different experiments and even between species.

**Availability:** FYPO releases are available from the Subversion repository at the PomBase SourceForge project page (https://sourceforge.net/p/pombase/code/HEAD/tree/phenotype_ontology/). The current version of FYPO is also available on the OBO Foundry Web site (http://obofoundry.org/).

**Contact:** mah79@cam.ac.uk or vw253@cam.ac.uk

## 1 INTRODUCTION

The fission yeast *Schizosaccharomyces pombe* is a eukaryotic model organism that has been used since the 1950s to study diverse biological processes including the cell division cycle, genome organization and maintenance, cell morphology and cytokinesis, signaling and stress responses, chromatin, gene regulation and meiotic differentiation (Egel, 2004). A large and active research community uses a wide variety of molecular genetic, cell biological and biochemical techniques to study *S.pombe*. With the completion of its genome sequence in 2002 (Wood *et al.*, 2002), fission yeast has also become amenable to genome-scale experimentation, and has emerged as a reliable model for studying processes involved in human disease and cell biology.

PomBase (http://www.pombase.org) has recently been established as a comprehensive model organism database that provides centralized access to information relevant to *S.pombe* (Wood *et al.*, 2012). PomBase encompasses a core of manual literature curation that provides detailed accurate curation of phenotypes, Gene Ontology (GO) annotations, genetic and physical interactions, protein modifications and many other types of data describing genes and their products. Manually curated data are supplemented by automatic gene annotation and large-scale datasets, and information about additional sequence feature types.

We define a phenotype as an observable characteristic, or set of characteristics, of an organism that results from the interaction of its genotype with a given environment. Extensive genetics research has been carried out using *S.pombe* over several decades, and a comprehensive set of high-quality curated phenotype data is in high demand in the *S.pombe* research community. A survey of *S.pombe* researchers conducted in 2007 identified phenotype annotation as the most requested feature not then available in a fission yeast database.

In response to community demand, we have developed the Fission Yeast Phenotype Ontology (FYPO), a formal ontology of phenotypes observed in fission yeast that will allow PomBase to provide consistent computable descriptions of phenotype data. Using FYPO, we have begun to curate accurate and detailed annotations of mutant allele phenotypes, with the aim of providing comprehensive coverage of phenotypes reported in the fission yeast literature. FYPO annotations are available on PomBase gene pages, and we envisage that the availability of genome-scale phenotype datasets will make new types of data analysis possible. Facilitated by the formal structure of FYPO, phenotype annotations can be shared and integrated with additional data, including other types of data obtained in fission yeast as well as phenotype data from other species.

## 2 APPROACH

The application of ontologies to biological curation has become widespread, and is best illustrated by the GO project (http://www.geneontology.org) (The Gene Ontology Consortium 2000, 2012), a collaborative effort to construct and use controlled vocabularies to support functional annotation of genes and their products in a wide variety of organisms. Ontologies facilitate consistent unambiguous descriptions of biological concepts, and can accommodate content at different levels of taxon specificity. Ontologies allow annotations at different levels of granularity, depending on what is known or what can be inferred, and provide mechanisms for quality control, consistency checking and error correction using collected data (both within and between ontologies). We sought to make these advantages available to curators and database users of PomBase phenotype annotations.

GeneDB *S.pombe* (http://old.genedb.org/genedb/pombe/), the predecessor database to PomBase, offered an extensive set of GO annotations, but did not use ontologies to capture other data types. GeneDB provided minimal phenotype annotation using a small, manually constructed, controlled vocabulary. The phenotype vocabulary was a flat list of ∼200 text descriptions, with no connections between the different descriptions. Furthermore, because the GeneDB phenotype vocabulary was designed and used exclusively for *S.pombe* annotations, it did not support any data sharing or integration between species or databases.

The launch of PomBase presented an opportunity to create an improved system for phenotype description, starting with a ‘blank slate’ and unconstrained by the limitations of the GeneDB vocabulary and annotation system.

### 2.1 Ontology design considerations

#### 2.1.1 The entity–quality model

We have constructed FYPO as a modular ontology that uses several existing ontologies from the open biological and biomedical ontologies (OBO) collection (Smith *et al.*, 2007) as building blocks to support the creation and maintenance of an extensive set of pre-coordinated phenotype descriptors. Terms from OBO ontologies, including the phenotypic quality ontology PATO (Gkoutos *et al.*, 2009), the GO, the Cell Ontology (CL; Meehan *et al.*, 2011) and Chemical Entities of Biological Interest (ChEBI) (de Matos *et al.*, 2010), are used to construct logical definitions for FYPO terms. For a phenotype, a logical definition follows the entity–quality (EQ) model (Mabee *et al.*, 2007): the entity is what is affected, and can be the whole cell, a population of cells, a part of a cell (corresponding to a GO cellular component) or an event such as a molecular function or biological process (represented by GO terms). An entity specification can be further refined with additional details using GO or ChEBI terms. The quality describes how the entity is affected, and is captured by a PATO term.

#### 2.1.2 Evaluation of available ontologies and term composition

Before commencing FYPO development, we examined the phenotype ontologies listed with the OBO Foundry, of which the Ascomycete Phenotype Ontology (APO; http://www.yeastgenome.org/cache/PhenotypeTree.html), developed for *Saccharomyces cerevisiae* (budding yeast) (Engel *et al.*, 2010) and other fungi, most closely matches FYPO in scope and intended application. Our evaluation was guided by the requirements of our highest-priority phenotype ontology applications. Most importantly, we require an extensive set of pre-composed phenotype terms for community annotation and querying.

Existing phenotype ontologies typically use one of two approaches: In a pre-coordinated (or pre-composed) ontology, such as the Mammalian Phenotype Ontology (Smith and Eppig, 2009), phenotype descriptions are composed in advance (i.e. separately from the annotation procedure). In other systems, such as *Dictyostelium discoideum* (Fey *et al.*, 2009) and *Danio rerio* (zebrafish) (Bradford *et al.*, 2011), phenotype descriptions are post-coordinated (post-composed) at the time of annotation; a curator chooses an entity and quality, and in some cases additional details, in parallel.

Phenotype annotation is one of the key features of PomBase’s newly developed community curation system (Rutherford *et al.*, manuscript in preparation), which allows researchers to contribute annotations from their publications directly to the database. For use by bench biologists, the simpler procedure of annotating to a single pre-composed term is more intuitive than the parallel annotation process required with post-composition. We also anticipate that biologists will wish to annotate to highly specific terms, making the reasoning supported by logical EQ definitions essential for ontology maintenance.

Annotations using APO terms, however, fall into the post-composed category: terms representing qualities and ‘observables’ are combined by curators as part of the annotation procedure. Thus, although phenotype descriptions using APO are conceptually compatible with the EQ model, entity–quality combinations are not incorporated into APO itself, nor do APO terms include logical definitions. Finally, curators using APO often add details drawn from other sources, including separate controlled vocabularies, meaning that much of the specific information captured in APO annotations is not incorporated into the ontology.

An additional ontology design consideration reflects distinctive features of fission yeast biology. *S.pombe* represents an early-diverging lineage within the Ascomycota (Taphrinomycota, formerly also known as Archiascomycetes) (James *et al.*, 2006). To accurately and consistently describe fission yeast phenotypes, we could reasonably expect to need specific terms that would not apply to the other ascomycete fungi (Saccharomycotina and Pezizomycotina) that have been annotated using APO. A new ontology offers maximal freedom to fit fission yeast-specific terms into a more general framework, without extensively restructuring an already-deployed vocabulary.

For these reasons, we have opted to develop FYPO independently despite the similarity in scope to APO.

### 2.2 Ontology content

#### 2.2.1 High-level organization

At the broadest level of classification, FYPO organizes terms along three axes. One axis distinguishes normal from abnormal phenotypes, where ‘normal’ is operationally defined as indistinguishable from characteristics of cells isogenic to the sequenced wild-type strain (972 h−), and ‘abnormal’ as detectably different from wild type, under the conditions in which a phenotype is assessed in a particular experiment.

A second axis classifies phenotypes by the entity affected; the broad categories correspond to effects on biological processes (as defined in GO), molecular functions (GO) or cellular structures (corresponding to GO cellular components). The third axis distinguishes phenotypes relevant at the level of a cell are from those that can be observed only in a population of cells.

Table 1 shows the top-level classifications in FYPO, with the numbers of *is_a* descendants and cumulative annotations for each term.

**Table 1.** Top-level terms in FYPO

Term name	ID	*is_a* descendants	Annotations
Abnormal phenotype	FYPO:0001985	1413	2546
Normal phenotype	FYPO:0000257	348	735
Cell phenotype	FYPO:0000002	1749	10 323
Cell population phenotype	FYPO:0000003	316	7584
Biological process phenotype	FYPO:0000300	1248	3604
Molecular function phenotype	FYPO:0000652	201	195

*Note*: For each term, the name and unique ID is shown, along with the number of terms that are its *is_a* descendants. The final column shows the number of individual annotations to the term or any of its descendants. (Data as of April 22, 2013.)

#### 2.2.2 Representing common types of phenotype

Further classification of the phenotype terms in FYPO, and their logical definitions, reflects several general categories into which phenotypes fall.

Some phenotypes, such as cell morphology, affect the entire cell (represented by the root of the Cell Ontology, CL:0000000). Morphological changes are also observed at the subcellular level, corresponding to GO cellular component terms. Phenotypes that affect cell size or shape refer to morphology qualities from PATO, as do phenotypes involving aberrant subcellular structures.

The largest category of phenotypes are those that affect (or inhere in) an entity corresponding to a GO biological process, i.e. phenotypes in which a cellular process does not proceed exactly as in wild-type cells. A smaller, but conceptually similar, group includes phenotypes that affect GO molecular functions such as binding or enzymatic activities. Phenotypes that affect biological processes and molecular functions refer to the corresponding GO terms, combined with PATO terms describing the alteration, e.g. ‘abolished’, ‘delayed’, ‘advanced’ (onset) or increased or decreased rate or frequency of occurrence.

Growth of cells on plates or in liquid medium is often evaluated, and changes in growth under specific conditions is taken to represent sensitivity (as in the case of cell death or decreased growth rate or yield) or resistance (unchanged or increased growth rate or yield) to a stimulus. Sensitivity to various chemicals can be modeled by combining the PATO term ‘increased sensitivity of a process’ with the GO biological process ‘vegetative growth of a single-celled organism’ and a ChEBI term representing the substance. Resistance to a chemical follows the same model, using PATO ‘decreased sensitivity of a process’. Sensitivity and resistance to stimuli other than chemical substances follow a similar pattern, but refer to GO terms for cellular responses to the stimuli. For example, ‘sensitive to osmotic stress’ (FYPO:0000270) refers to the GO term ‘cellular response to osmotic stress’ (GO:0071470).

In addition to PATO, GO and ChEBI, FYPO draws on the Sequence Ontology (SO) (Eilbeck *et al.*, 2005) for a small number of terms that refer to specific DNA or RNA sequence regions, a small number of Cell Ontology (CL) terms to distinguish phenotypes that affect vegetatively growing cells or spores and a single term the BRENDA Tissue Ontology (BTO) (Gremse *et al.*, 2011) is used for phenotypes that depend on, or affect, the growth medium.

Table 2 summarizes the usage of OBO ontology terms in FYPO.

**Table 2.** Usage of ontology terms in FYPO logical definitions: of 2010 total FYPO terms (as of April 22, 2013), 1802 have logical definitions

Ontology	Unique external ontology terms	FYPO terms
BRENDA tissue/enzyme (BTO)	1	91
Chemical entities of biological interest (ChEBI)	196	480
Cell ontology (CL)	3	236
Gene ontology (GO)	570	1880
Phenotypic quality (PATO)	88	1709
Sequence ontology (SO)	10	31

*Note*: The table shows the external ontologies used in FYPO logical definitions. ‘Unique external ontology terms’ denotes the number of different terms from the indicated ontology that are used; ‘FYPO terms’ indicates the number of FYPO terms that have a logical definition using one or more terms from the indicated ontology.

#### 2.2.3 Phenotype modeling challenges

FYPO also includes a number of terms that do not fit the simple logical models described above. The principal types are complex phenotypes, which encompass more than one quality, and phenotypes that affect cell populations.

**Fig. 1.**
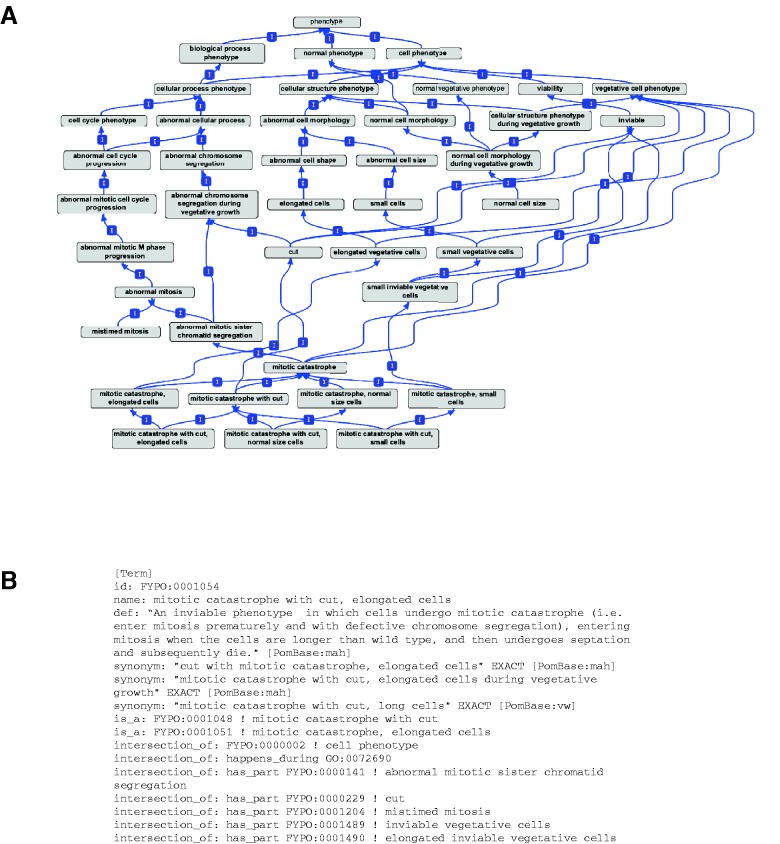
Several specific types of mitotic catastrophe have been defined based on whether cell size or shape is affected, and whether the cells undergo septation despite the failure of chromosome segregation (‘cut’ phenotype). As the different specific mitotic catastrophe phenotypes support different interpretations of the underlying biology, the distinctions among these related phenotypes are valuable for downstream applications of phenotype annotations. (**A**) Graphical view of terms and *is_a* relationships, which classify the terms. More specific terms build on less specific terms by addition of differentiating features. A complex phenotype such as ‘mitotic catastrophe with cut, elongated cells’ has multiple paths to the root (most general term) of the ontology via different parents, allowing annotations at any level of specificity. Also note that the paths in FYPO parallel the paths describing mitosis and the cell cycle in GO as well as those in the cell morphology area of FYPO. (**B**) OBO stanza defining FYPO:0001054 ‘mitotic catastrophe with cut, elongated cells’. Note that the two *is_a* relationships shown are manually asserted. The logical definition is specified by the intersection_of lines, and the def line provides a human-readable definition

Complex phenotypes can be represented as having simpler phenotypes as parts. To illustrate, Figure 1 shows the portion of FYPO describing ‘mitotic catastrophe’ phenotypes, which arise when defects in mitotic chromosome segregation lead to cell death. The most general mitotic catastrophe term (FYPO:0001047) is defined as the combination of ‘inviable’ (FYPO:0000049) with ‘abnormal mitotic sister chromatid segregation’ (FYPO:0000141). Because mitotic catastrophe may occur with one or more additional features such as altered cell shape or size or a ‘cut’ phenotype (i.e. septation despite abnormal chromosome segregation), several more specific terms are included, and their logical definitions specify the additional parts. ‘Mitotic catastrophe with cut’ (FYPO:0001048), for example, has the additional parts ‘cut’ (FYPO:0000229) and ‘mistimed mitosis’ (FYPO:0001204), whereas ‘mitotic catastrophe, elongated cells’ (FYPO:0001051) adds FYPO:0001204 and ‘elongated vegetative cells’ (FYPO:0001122). All biologically relevant combinatorial possibilities can be built, including ‘mitotic catastrophe with cut, elongated cells’ (FYPO:0001054).

Although most fission yeast phenotypes can be represented as properties of a cell (including events taking place in a cell), some phenotypes can only be observed at the level of a population. These cell population phenotypes reflect properties of what cells do in groups, and pose particular challenges for logical modeling because they do not represent characteristics of a single organism. Some examples are colony morphology (‘abnormal colony morphology’ FYPO:0000150), flocculation (‘flocculating cells’ FYPO:0000155) and filament morphology. Some cellular processes can also be studied in cell populations, giving rise to population-level phenotype observations; one example is septation, for which the ‘septation index’, i.e. the proportion of cells in a population observed undergoing septation under given conditions is often measured. Although FYPO:0000155 is defined as ‘increased occurrence’ (PATO:0002051) of ‘flocculation’ (GO:0000128), most cell population phenotype terms are among the small fraction in FYPO that do not yet have logical definitions.

To accurately model some phenotypes has required the introduction of a few relations that are not defined in the OBO Relations Ontology (RO; http://code.google.com/p/obo-relations/) or the Basic Formal Ontology (BFO; http://www.ifomis.org/bfo/) at present. Some, such as *during* and its subtypes *exists_during* and *happens_during*, which are used to link process or structural phenotypes to periods such as cell cycle phases, are borrowed from a set of relations developed by the GO Consortium for its annotation extensions (Huntley *et al.*, manuscript in preparation). Others, such as *includes_cells_with_phenotype*, which links cell population phenotypes with cell-level phenotypes of cells within the population, have been created specifically for FYPO and will be submitted as candidates for addition to RO.

#### 2.2.3 Current advantages of FYPO usage

FYPO’s modular structure and formal logical definitions confer a number of advantages, as specified below:

In ontology development, it is feasible to manage a large set of terms, to define phenotypes precisely and to represent phenotype descriptions with complex relationships to each other and to underlying biological phenomena. Reasoning software can use FYPO’s logical definitions to infer links between terms and to detect redundancy and other errors, which streamlines ontology development. Furthermore, because the definitions refer directly and specifically to terms from other OBO ontologies, reasoning over FYPO also keeps its structure consistent with external ontologies such as GO, ChEBI and PATO. Text details are also more easily managed in FYPO than in a flat, manually managed, list. For example, synonymous words and phrases can be included to aid querying. Minor inconsistencies, such as misspellings and duplications, are easily avoided.

In addition to facilitating ontology development and quality control, FYPO supports much more effective manual curation than the legacy vocabulary from GeneDB. With many more specific terms, annotators can capture much richer, more detailed phenotype information. The text and logical definitions help annotators maintain accuracy and consistency in using a large set of ontology terms.

Both the increased specificity and the structure of the ontology also support sophisticated querying and computational analysis.

**Fig. 2.**
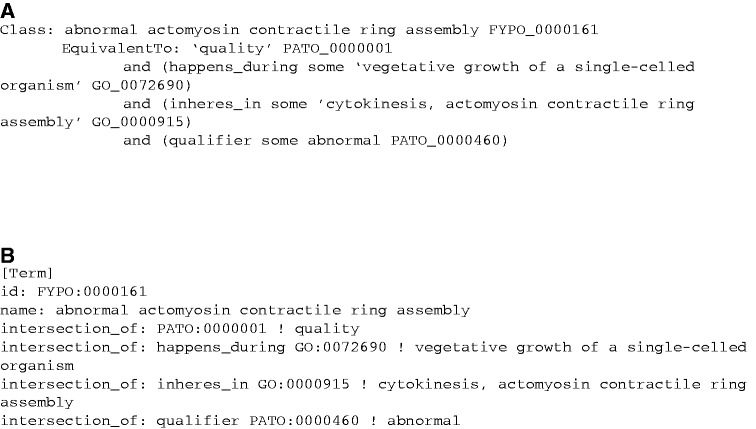
Representation of FYPO:0000161, ‘abnormal actomyosin contractile ring assembly’, and its logical definition. (**A**) Manchester syntax. (**B**) OBO format

Terms and annotations relevant to cytokinesis phenotypes illustrate many of the improvements that FYPO has facilitated. This topic is one of a number in which the GeneDB vocabulary had a general descriptor such as ‘phenotype, cytokinesis defects’ included as a substring of more specific entries such as ‘phenotype, cytokinesis defects, contractile ring, absent’, but the terms were not otherwise related. Although a text search for the more general string would find both terms, a search for genes annotated to the general term would not retrieve genes annotated to the more specific term. In contrast, the FYPO term ‘abnormal actomyosin contractile ring assembly’ (FYPO:0000161) has a logical definition that states that the quality ‘abnormal’ (PATO:0000460) *inheres_in* the process of actomyosin contractile ring assembly during cytokinesis (GO:0000915,’cytokinesis, actomyosin contractile ring assembly’). (*Inheres_in* formally states that the PATO quality is an attribute of the GO process or other affected entity.) Figure 2 shows the logical definition for FYPO:0000161 in Manchester syntax and OBO format. The classification of the phenotype term in FYPO parallels that of the biological process term in GO, in which ‘actomyosin contractile ring assembly’ is both a type of ‘actin cytoskeleton organization’ and a part of ‘cytokinesis’. Any mutant alleles annotated to FYPO:0000161 can therefore be retrieved by queries for mutations that affect the actin cytoskeleton as well as those affecting cytokinesis.

Normal phenotypes are represented in FYPO using the same logical structures, and at the same level of detail, as abnormal phenotypes. Fission yeast is particularly amenable to normal phenotype annotation because the commonly used laboratory strains are all isogenic, making unambiguous recognition of normal, and therefore also abnormal, characteristics straightforward. Annotation of normal phenotypes allows curators to document mutations that cause no phenotypic changes with respect to certain assays, or under standard growth conditions. This provides important, albeit negative, information about gene function, and makes the total set of fission yeast phenotypes more comprehensive. For example, deletion of the small GTPase Ras1 causes defects in conjugation (mating) and sporulation (Herskowitz, 1995; Hughes *et al.*, 1990; Papadaki *et al.*, 2002). Deletion of the Ras1-activating guanyl nucleotide exchange protein Ste6, however, causes defects only in conjugation. The normal sporulation phenotype observed in the *ste6* null mutant indicates that Ras1 must have other regulators and downstream effectors besides Ste6.

## 3 METHODS

FYPO is built using OBO-Edit (Day-Richter *et al.*, 2007). Logical definitions are constructed in OBO-Edit for each term that can be represented as described in the PATO XP best practices (http://obofoundry.org/wiki/index.php/PATO:XP_Best_Practice). In particular, FYPO uses *inheres_in* both for qualities of processes and physical entities, as is common in other related efforts (Mungall *et al.*, 2010). It has since transpired that future versions of BFO may prohibit this usage, in which case we will either modify the pattern we use, or use a broader relation, which will be incorporated in RO (C.J.Mungall, personal communication). The initial set of FYPO terms was based on a set of 208 free-text descriptors used to annotate deletion (null) phenotypes in GeneDB. Additional terms were generated by combining a PATO quality with GO terms frequently used in *S. pombe* annotations supported by phenotypic evidence (using the evidence ‘inferred from mutant phenotype’, IMP). In ongoing FYPO development, terms are added or modified as needed to describe phenotypes in published literature accurately and precisely. Term requests may come from PomBase curators or community researchers. Regular releases of FYPO are generated using the OBO Ontology Release Tool (Oort; http://code.google.com/p/owltools/wiki/Oort), and include OBO and OWL formats. Reasoning uses the ELK reasoner (Kazakov *et al.*, 2011) as part of the Oort release process. Links inferred by the reasoner during the Oort release are reviewed periodically, and any ontology errors that cause anomalous inferences are corrected.

## 4 DISCUSSION

We have developed a formal ontology of phenotypes observed in fission yeast, which now includes over 1900 terms, to support phenotype curation in PomBase.

### 4.1 Applications of FYPO

The primary application of FYPO is to provide the phenotype information demanded by the *S.pombe* research community, initially in the form of annotations displayed on PomBase gene pages, detailing alleles, type of supporting evidence, and literature citations as well as FYPO terms. At present all fission phenotype annotation is supported by published experimental data which has been manually curated. To date, over 6000 legacy annotations have been converted from the GeneDB controlled vocabulary to FYPO terms, and a comparable number of new annotations have been curated.

Enhanced phenotype description using FYPO supports curation of both classical low-throughput and emerging high-throughput experiments. The latter will become increasingly important as researchers use the genome-wide deletion collection that has recently become available; genome-wide viability data are published (Kim *et al.*, 2010) and many more comprehensive phenotype screens are possible. We also include phenotype curation in the new community curation tool (Rutherford *et al.*, manuscript in preparation), which enables us to incorporate phenotype annotations, along with supporting data on alleles and experimental conditions, directly from expert researchers. Moreover, because users can request new phenotype terms, community contributions offer substantial benefits to the phenotype ontology itself as well as the collection of *S.pombe* phenotype annotations.

As high-throughput experiments become more common, and manual curation of phenotypes from small-scale experiments becomes more complete, the body of *S.pombe* phenotype data will become sufficiently comprehensive to support enrichment analyses analogous to those routinely performed using GO annotations (for example, see Deshpande *et al.*, 2009; Helmlinger *et al.*, 2008; Kim *et al.*, 2010; Marguerat *et al.*, 2012; Shimanuki *et al.*, 2007; Xue-Franzén *et al.*,2006). We anticipate that comprehensive phenotype annotation, and analyses thereof, will complement GO annotation data. PomBase curators have begun reviewing GO annotations based on mutant phenotypes, to remove those that are known to represent indirect ‘downstream’ effects. Many experimenters, however, will likely want to include both direct and indirect effects when analysing processes over- or under-represented in gene sets. The use of phenotype annotations to capture indirect effects, combined with GO annotations representing direct effects, allows us to maintain the direct–indirect distinction while supporting comprehensive enrichment analyses. As an example, a number of genes, such as the endoplasmic reticulum calcium-transporting ATPase Cta4, the pantothenate transporter Liz1, the mitochondrial DNA polymerase Pog1 and the DNA replication factor A subunit Ssb1, have annotations to FYPO terms describing cytokinesis defects, but are not annotated to cytokinesis in GO. FYPO annotations will also provide access to statistical over-representation of gene lists for cellular phenomena that fall outside the scope of GO (such as drug sensitivity, cell shape defects, cell lysis, etc.). We further speculate that sets of mutants will emerge with the same phenotypic signatures but distinct GO categories, which would suggest that distinct sets of proteins may be involved (directly or indirectly) in the same cellular processes; such features would not be immediately evident from GO annotation alone.

### 4.2 Logical structure and data integration

We have opted to pre-compose FYPO terms, primarily to simplify the annotation process. Although pre- and post-composed terms may be semantically equivalent, the parallel annotation process required with post-composition is not well suited to community curation. The simpler procedure of annotating to a single pre-composed term is more intuitive for bench biologists.

Because >90% of FYPO terms have logical EQ definitions, however, we can also realize the benefits of explicit references to other OBO ontologies and reasoning. Notably, FYPO is compatible with the Cell Phenotype Ontology (CPO) (Hoehndorf *et al.*, 2012), a species-neutral ontology of morphological and physiological phenotypic characteristics of cells, cell components and cellular processes that supports automated synchronization with GO and integration of cellular phenotype data across species. Like CPO, FYPO defines many phenotypes at the cellular level, in terms of cellular processes or structures (both referring to GO) and how they are affected (referring to PATO). The shared aspects of phenotype representation mean that FYPO will be able to take advantage of CPO’s automated synchronization to maintain consistency with GO and PATO. Conversely, the inclusion of FYPO and its associated fission yeast phenotype annotations provide CPO with a set of high-quality data representing an important model organism, enriching its integrated datasets.

On a related note, the developers of the Ontology of Microbial Phenotypes (OMP; http://microbialphenotypes.org/) are taking a similar approach to construct EQ-based descriptions of phenotypes observed in microorganisms, especially in *Escherichia coli*. As considerable overlap in scope is likely between OMP and FYPO, the common underlying ontology structure will facilitate possible future integration of ontology terms or annotation data. Because EQ model-based phenotype integration methods can be used to align pre- and post-composed phenotype terms (Mungall *et al.*, 2010), we can also explore ways to align APO with FYPO to improve sharing of phenotype descriptions and annotation data.

### 4.3 Future work

As manual curation of phenotypes continues, we will add terms to FYPO as required, and we will explore ways to improve formal phenotype representations. For example, we anticipate that the recently launched Population and Community Ontology (PCO; http://code.google.com/p/popcomm-ontology/) will provide terms that can be incorporated into logical definitions for FYPO population phenotypes.

We also envision extending FYPO to accommodate high-throughput experiments. For example, we will add complex phenotypes such as whole-transcriptome signatures used for expression quantitative trait locus mapping. High-throughput screens will also capture quantitative data associated with phenotypes such as growth rates, survival rates following stress, or cell size and shape. Few of the challenges of modeling quantitative phenotypes have been met among the broader community of ontology developers working on phenotype representation, but we will work with both *S.pombe* researchers and ontology developers to meet emerging community needs.
